# Exploring the correlation between suicide and homicide rates with percentage of gentrification and by sex

**DOI:** 10.3389/fsoc.2026.1788950

**Published:** 2026-03-20

**Authors:** Thelma Beatriz González-Castro, Alma Delia Genis-Mendoza, Carlos Alfonso Tovilla-Zárate, María Lilia López-Narváez, Ana Fresán, Cindy Alejandra García Juárz, José Antonio Ovando-Ricardez, Jorge Luis Hernández Vicencío, Yazmín Hernández-Diaz, Humberto Nicolini

**Affiliations:** 1 División Académica Multidisciplinaria de Jalpa de Méndez, Universidad Juárez Autónoma de Tabasco, Jalpa de Méndez, Mexico; 2 Laboratorio de Genómica de Enfermedades Psiquiátricas y Neurodegenerativas, Instituto Nacional de Medicina Genómica, Ciudad de México, Mexico; 3 División Académica Multidisciplinaria de Comalcalco, Universidad Juárez Autónoma de Tabasco, Comalcalco, Mexico; 4 Dirección de Investigaciones Biomédicas en Salud Mental, Instituto Nacional de Psiquiatría Ramón de la Fuente Muñiz, Ciudad de México, Mexico; 5 Universidad Tecnológica de Tabasco, Comalcalco, Mexico

**Keywords:** gentrification, homicide, Mexico, sex, suicide

## Abstract

**Introduction:**

Gentrification is present around the world. Little of suicide and homicide has been studied in relation to gentrification. The objective of this study was correlate the suicide and homicide rates with the percentage of gentrification by gender and by municipalities of Mexico City.

**Methods:**

This descriptive and observational study collected data from suicide and homicide of the Attorney General’s Office of Mexico City, Mexico to calculate the suicide and homicide rate in Mexico City, Mexico. The gentrification percent by municipalities of Mexico City were taken from Airbnb platform. Spearman’s correlation and scatterplots were created to show suicide correlation between gentrification percentage and suicide and homicide.

**Results:**

A significant negative correlation was obtained between gentrification percentage and suicide (r= -0.51, *p*=0.04). Also, a significant negative correlation was observed between gentrification percentage and suicide in women (r= -0.58, *p*=0.01). Indicative that in municipalities when the gentrification increases, suicide rate decreases. No correlations were observed between homicide and gentrification percentage by municipalities or by sex.

**Discussion:**

Our results show that in Mexico City, the gentrification percentage is negatively correlated with suicide rates, but it is not correlated with homicide rates. This suggests that gentrification could be linked to the prevention of suicide especially in women. However, further studies are needed to achieve conclusive results.

## 1 Introduction

Gentrification is the process of transforming an underdeveloped or low-potential area into a more attractive residential, cultural, and commercial space through financial investment for new residents in the short or long term (Anguelovski et al., 2021). It has been estimated that gentrification is present for the restructuring of social relations in spaces, centered on sectors or boroughs of the city with greater economic capacity. It is characterized by the remodeling of homes, spaces, buildings, and new businesses that favor the entry of higher-income groups and a larger population. Gentrification has brought positive aspects worldwide, such as improved transportation infrastructure, services, and employment opportunities, among others (Zuk et al., 2017). However, it also brings negative aspects, including increased tensions between new and long-term residents, the loss of cultural establishments and small businesses, the decline of green spaces, and, above all, the rising cost of living and housing (Anguelovski, 2015; Oscilowicz et al., 2020). Growing evidence suggests that gentrification is linked to mental health outcomes (Alroy et al., 2023; Torres, 2020; Wood et al., 2023). While the literature has documented negative mental health impacts among residents exposed to neighborhood change (gentrification), others have reported mixed or context-specific associations (Pineault et al., 2025; Youngbloom et al., 2023).

Mexico City is the Mexican capital and the country’s largest urban center, and it is steeped in history, culture, and traditions. Mexico City has undergone multiple transformations. These modernizations are associated with restructuring processes characterized by pro-business urban management, contributing to the phenomenon of gentrification (Olivera and Delgadillo, 2014). This generates greater demand in certain municipalities of Mexico City (López et al., 2020).

Suicide and homicide in Mexico City have increased. In 2024, the suicide rate in Mexico City was of 6.8 per 100,000 persons. Whereas in 2014 and 2019 they were 5.1 and 5.6 per 100,000 people, respectively (INEGI, 2025b). Similarly, the rate of homicide was 24.9 per 100,000 people in 2023 and 25.6 per 100,000 people in 2024 (INEGI, 2025a). Furthermore, suicide represents a critical mental health outcome influenced by multifactorial determinants, including socioeconomic conditions, security conditions among other factors. Hence, some studies demonstrate a plausible mechanism implicated in standard measures of gentrification and some violent acts such as suicide and homicide (Baranyi et al., 2021). Mexico City’s unique heterogeneity encompasses a variety gentrifying neighborhoods, which provides an important opportunity to investigate spatial patterns of suicide and homicide in relation to gentrification (Aguilar-Velázquez et al., 2024). Consequently, the aims of this study are (a) to know the suicide and homicide rates in the municipalities of Mexico City; (b) correlate the suicide and homicide rates with percentage of gentrification in the municipalities of Mexico City; and (c) to compare the homicide and suicide rates by percentage of gentrification and by gender.

## 2 Materials and methods

### 2.1 Study design

This is a descriptive, observational and retrospective study. Data and information were collected from the Attorney General’s Office (AFO) of Mexico City. AFO is responsible for determining the causes of death; as well as categorizing the causes of death as homicide or suicide. Cases with incomplete information or those not properly categorized were excluded. Data from all the municipalities of Mexico City were included. The data are free of the Attorney General’s Office of Mexico City (Mexico FGDJCD, 2025). Only data of 2024 year were collected. The data were accessed until 01/11/2025.

### 2.2 Homicide, suicide, and gentrification percentage

Homicide. Attorney General’s Office included the categories of homicide. Categories such as homicide by firearm, blunt force trauma and intentional homicide were included. Additionally, suicide categories were included. The Attorney General’s Office included only two categories for death by suicide: suicide and death on the subway. The suicide and homicide rates by municipalities of Mexico city, were calculated from the results of the Population and Housing Census conducted by the National Institute of Statistics and Geography (INEGI) (Geografia INDEY, 2025) and the main demographic indicators (population projections) provided by the National Population Council (CONAPO) (Poblaciones CND, 2025). The suicide and homicide rates were calculated for municipalities based on per 100,000 people.

Gentrification percentage. We used the percentage of gentrification according to the literature on gentrification in Mexico City (Reyez, 2025). Our methodology was based on the housing dataset provided by the Airbnb platform (Airnbnb, 2025; Sequera, 2025). This dataset contains information on 25,623 entire houses or apartments, private or shared rooms, and recently added hotel rooms as well in Mexico City in 2025. We focused on the location of these spaces to determine which borough has the highest rate of gentrification. The Airbnb database was accessed until 01/11/2025.

### 2.3 Statistical analysis

Gentrification percentage and rates of suicide and homicide were considered as continues variable. Then, *t*-test were used for comparing differences between rate of suicide or homicide between males and females. We used the Spearman’s correlation to measure the correlation between gentrification percentage and rates of suicide or homicide in the general population and by sex. We measure statistical power of the present study using the G*Power 3.1 software. We used tail two, rho = 0.5, *α* err pro = 0.05, Total sample size: 16, H0 = 0 and a power = 0.53. Finally, scatterplots were created to show suicide, homicide rates and gentrification percentage using the R programming language (R 4.5.2). Data preprocessing and manipulation were carried out with tidyverse packages, specifically dplyr (v1.1.4) and tidyr (v1.3.2), and visualization was produced using ggplot2 (v4.0.0), according to the study variables.

## 3 Results

### 3.1 Gentrification percentage in the city and municipalities

Table 1 shows the gentrification percentage and rates of suicide and homicide by municipalities of Mexico City. The gentrification percentage in Mexico City was 26.40% in 2024. The highest rates of gentrification were found in the Cuauhtémoc and Miguel Hidalgo municipalities, with percentages of 44.6 and 16.4%, respectively Table 1. Tláhuac y Milpa Alta were the municipalities with lower gentrification with 0.2 and 0.1%, respectively.

**Table 1:** Rates of suicide, homicide and gentrification percentage.

Municipalities	Gentrification percent	Suicide rates*			Homicide rates**		
		All	Male	Female	All	Male	Female
Alvaro Obregón	3.5	3.76	5.71	1.98	11.56	20.39	2.23
Azcapotzalco	1.4	4.29	6.20	2.57	12.64	21.95	2.99
Benito Juárez	11.2	3.38	3.38	1.69	6.76	12.55	1.69
Coyoacán	6.4	5.61	7.01	3.43	11.23	18.94	3.12
Cuajimalpa	1.5	5.32	9.19	1.71	5.32	8.27	2.57
Cuauhtemoc	44.6	5.75	9.67	1.42	21.54	38.68	5.35
Gustavo A Madero	2	3.72	6.27	0.83	11.07	18.82	3.51
Iztacalco	1.7	3.73	5.75	1.42	13.20	23.01	4.28
Iztapalapa	1	4.80	7.85	1.71	15.85	27.67	4.50
Magdalena Contreras	0.5	8.89	14.31	3.88	7.68	15.15	0.77
Miguel Hidalgo	16.4	4.80	8.20	1.35	8.64	15.89	2.25
Milpa Alta	0.1	7.57	11.51	3.73	10.09	15.35	2.49
Tláhuac	0.2	7.34	11.97	2.96	15.45	26.56	4.44
Tlalpan	2.8	3.93	6.42	1.62	11.94	21.91	2.16
Venustiano Carranza	3.3	4.77	7.24	0.85	17.28	28.49	6.87
Xochimilco	0.5	5.86	8.68	3.11	13.53	23.32	4.00

*t-test between male and female. t = 8.09, *p* ≤ 0.0001 **t-test between male and female. t = 9.56, *p* ≤ 0.0001.

### 3.2 Suicide rates and gentrification in the city and municipalities

**Figure 1:**
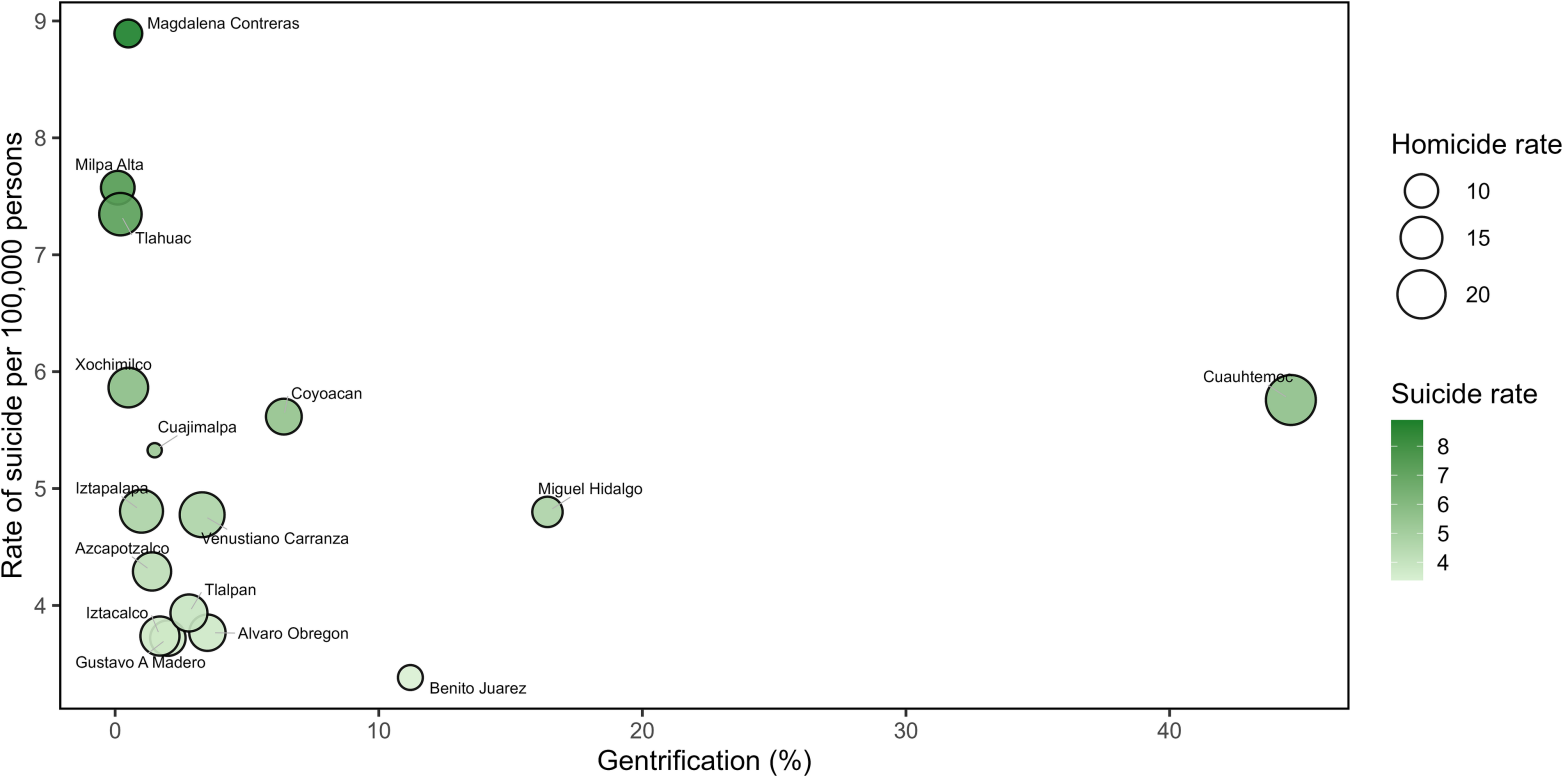
Comparison of homicide and suicide rates (per 100,000 inhabitants) and the percentage of gentrification in the general population of Mexico City in 2024, distributed by municipalities.

The suicide rate calculated in Mexico City was 4.81 cases per 100,000 inhabitants in 2024. Figure 1 shows the homicide and suicide rates per 100,000 inhabitants and the percentage of gentrification for the general population in Mexico City, distributed by municipalities. Milpa Alta, Tláhuac and Magdalena Contreras municipalities showed the highest incidence of suicide as the lowest percentage of gentrification (Figure 1).

### 3.3 Homicide rates and gentrification in the city and municipalities

The homicide rate calculated in Mexico City was 12.95 homicides per 100,000 inhabitants in 2024. Cuauhtémoc municipality showed a higher homicide rate and higher gentrification percentage (44.6%). This was followed by Venustiano Carranza and Iztapalapa municipalities, that showed a rate of homicides of 17.28 and 15.81 homicides per 100,000 persons and showed lower gentrification percentages (3.3 and 1% of gentrification) (Figure 1).

### 3.4 Suicide and homicide by gender

The rate of suicide and homicide by gender and by municipality are shown in Table 1. The suicide rate in males in Mexico City was 7.51 per 100,000 persons. Whereas suicide rate in females was of 1.90 per 100,000 persons. As can be seen, the rate is higher in males (t = 8.09, df = 30 *p* ≤ 0.0001). The homicide rate differed between sex; in males the rate was 22.62 per 100,000 persons, and in females of 3.55 per 100,000 persons (t = 9.56, df = 30 *p* ≤ 0.0001).

### 3.5 Correlation between suicide, homicide and gentrification

Finally, we looked for a correlation between gentrification and suicide or homicide. We found a significant negative correlation between gentrification and suicide (r = −0.51, *p* = 0.04). No correlation was observed between gentrification and homicide (r = −0.2, *p* = 0.93) (Table 2). In the analysis by sex, we observed significative negative correlation between gentrification and suicide in women (r = −0.58, *p* = 0.01), but non significative correlation was observed in men (Table 2).

**Table 2:** Correlations of gentrification and percentages and suicide or homicide.

Characteristic	All sample		Women		Men	
	*r*-value	*p*-value	*r*-value	*p*-value	*r*-value	*p*-value
Suicide	**−0.51**	**0.04**	**−0.58**	**0.01**	−0.47	0.06
Homicide	−0.02	0.73	−0.04	0.86	0.01	0.94

Statistical significance is bold.

## 4 Suicide, homicide and gentrification by women

In Mexico City in 2024, the homicide rate for women were 3.55 per 100,000 women. Whereas the suicide rate for women in Mexico City was 1.90 per 100,000 women.

**Figure 2:**
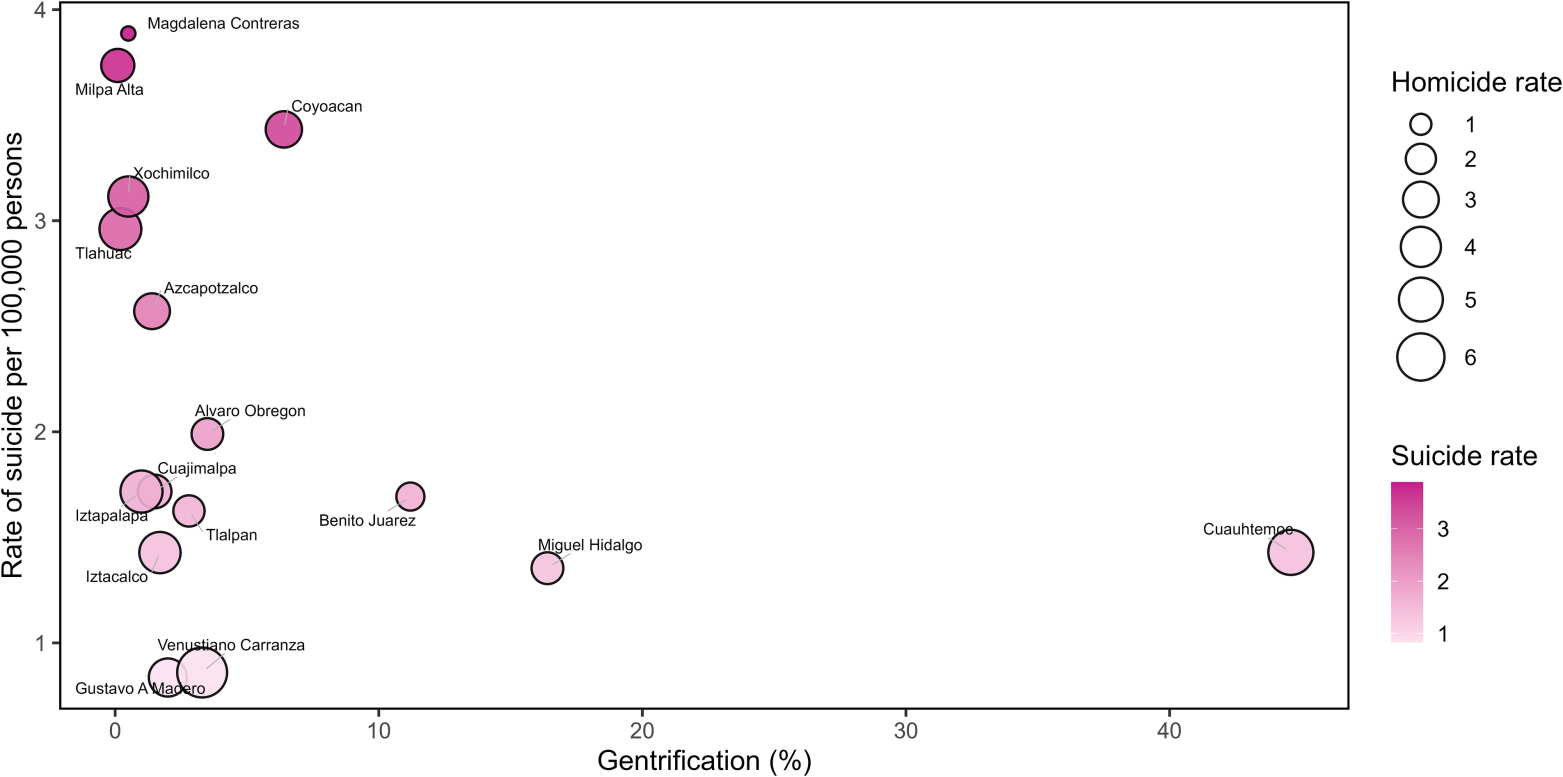
Comparison of homicide and suicide rates (per 100,000 inhabitants) and the percentage of gentrification in female population of Mexico City in 2024, distributed by municipalities.

The highest rate of suicide was observed in the Magdalena Contreras municipality (3.88 per 100,000 women). However, homicide rate in the same municipality was low at 0.77 per 100,000 women. This municipality is among the lowest percentages of gentrification (Figure 2). On the hand, Venustiano Carranza municipality had higher homicide rate (6.87 per 100,000 women), but lower suicide rate (0.85 per 100,000 women) and is among the lowest percentages of gentrification.

## 5 Suicide, homicide, and gentrification by man

**Figure 3:**
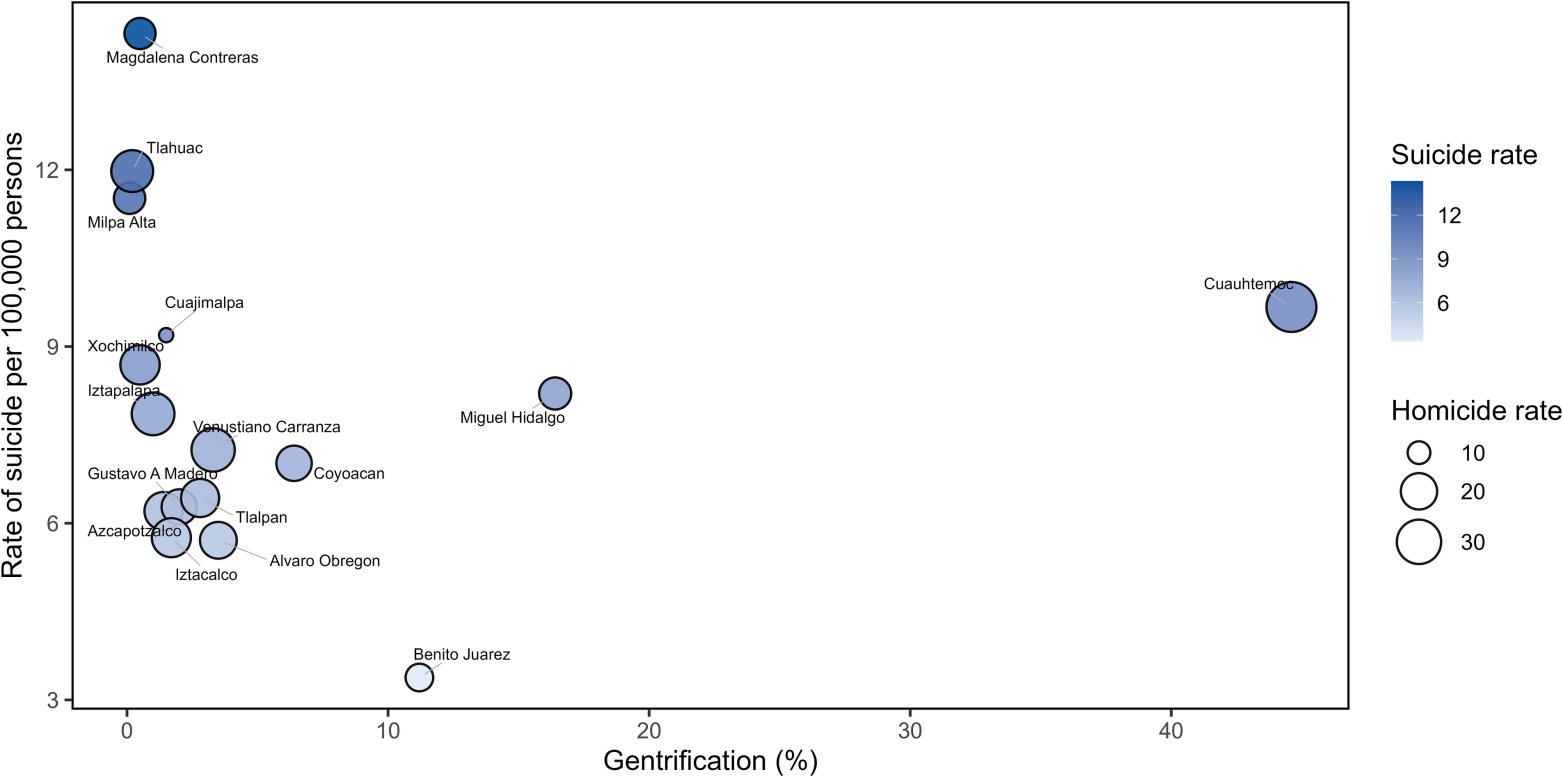
Comparison of homicide and suicide rates (per 100,000 inhabitants) and the percentage of gentrification in male population of Mexico City in 2024, distributed by municipalities.

The homicide rate for men in the Mexico City was 22.62 per 100,000 men in 2024. Whereas the suicide rate was 7.51 per 100,000 men. Benito Juarez municipality showed the lowest suicide rate (3.38 per 100,000 men) and is the third municipality with gentrification. In contrast, Magdalena Contreras municipality registered the highest suicide rate in 2024, at 14.31 per 100,000 men, a lower homicide rate and is the lowest municipality with gentrification. Finally, the Cuauhtémoc municipality had the highest homicide rate for men (38.68 per 100,000 men) and a moderate suicide rate of 9.67 per 100,000 men and is the municipality with highest percentage of gentrification (Figure 3).

## 6 Discussion

Over the last decade, gentrification has been proposed as a determinant factor in population health. Nevertheless, evidence indicated a complex association with mental health and neighborhood dynamics. A diversity of comprehensive reviews showed that gentrification has highlighted both positive and negative impacts on mental wellbeing. Therefore, our objective was to evaluate the relationship between gentrification and suicide and homicide rates in the municipalities of Mexico City.

Firstly, main outcome revealed that gentrification has a negative correlation in the suicide rates (*r*-value = −0.51; *p*-value = 0.04); especially in women (*r*-value = −0.58, *p*-value = 0.01). This suggests that greater gentrification is correlated with lower suicide rates. There are several factors that could be participating in this relationship. Based on prior literature, it has been documented economic status is vital to have a positive effect on physical or mental health as consequences of gentrification. An example is a study comparing marginalized Black residents and white residents with moderate-high profile status. White residents have a better response to gentrification, increasing health behavior (e.g., outdoor physical activity; Bhavsar et al., 2020). Moreover, previously published evidence showed that gentrifies and residents who identified with their neighborhoods experienced better mental health outcomes (Pineault et al., 2025). Hence, it is pivotal to take this into consideration when analyzing suicide rates. Gentrification processes are often accompanied by improvements in neighborhood, such as better security, revitalized public spaces among others that have been linked to overall mental health benefits. Especially in women a greater perception of safety and environmental influences on daily activities are involved with higher quality of mental health (Li et al., 2022). Therefore, the above could explain the decrease in suicides in municipalities with major gentrification. Furthermore, evidence from population-based studies suggests that improvements in urban infrastructure, such as better open and green space, are correlated with an improvement of mental health issues; which a key determinant of suicide risk (Barboza-Salerno et al., 2025). This suggest that an increase of gentrification allowed upgrade urban environments and better supportive infrastructure, which may contribute to lower suicide rates by improving mental health issues at the community level (Barboza-Salerno et al., 2025; Zayas-Costa et al., 2021).

In the other hand, although gentrification has been correlated with changes in crime patterns, in our analysis among Mexican Citizens, this relationship has been not observed. Homicide is interpersonal lethal violence, which is commonly driven by complex social, economic, and structural factors that extend far beyond neighborhood socioeconomic upgrading alone (Kim, 2019). Hence, local processes such as gentrification may have only a limited explanatory power when these factors are dominant (Li et al., 2022).

Although prior literature suggests that gentrification may enhance perceived safety, interestingly our data of Cuauhtémoc municipality showed both the highest gentrification percentage and elevated homicide rates. First, this data highlight the complexity of urban transformation processes, because perceived safety and homicide rates represent distinct constructs (Argueta et al., 2023). Concerning to perceived safety often it is linked with factors such as neighborhood upgrading, improved lighting, commercial activity, and increased police presence; meanwhile homicide rates are often associated with broader structural determinants such as organized crime, economic inequality, or high population mobility (Martin, 2025; Tehrani et al., 2025). In this sense, the Cuauhtémoc municipality is characterized by intense commercial activity, tourism, nightlife, and a large transient population, which may inflate homicide rates calculated per resident population (Baca-López et al., 2021).

Some limitations of this study should be acknowledged when interpreting the findings. First, Correlations were analyzed at the municipal level, which raises the possibility that the observed correlations may not reflect individual risk but rather contextual patterns. Second, the statistical power of the study may be limited. Because the analyses were conducted at the municipal level and the number of municipalities included was relatively small, the study may be underpowered to detect meaningful correlations or to support broader generalizations. Third, the analysis was based on rates measured at a single point in time. We did not examine temporal changes in suicide or homicide rates. However, we used the most recent data available published by the Mexican Attorney General’s Office to calculate suicide and homicide rates. Another point is that the analysis did not account for potential confounding variables, such as income inequality, employment, access to mental health services, among other some factors, which are known to influence suicide and homicide patterns in Mexico. Related to this, another point that we want to highlight is that we only could have access to Airbnb records. Hence data should be taken into consideration because there are other factors such as prior investment, pre-existing high income neighborhoods and other socio-economic advantages with a direct impact in gentrification. Therefore, with this missing data a prior, we could not assume that gentrification results in an improvement of infrastructure or other socio-economic features. Additionally, it is necessary to highlight that gentrification, and its potential mental health impacts may unfold in a gradual process over longer periods. Longitudinal studies are needed to assess whether changes in gentrification precede changes in suicide or homicide rates over time.

## 7 Conclusion

In conclusion, our findings indicate a significant negative correlation between gentrification and suicide rates, particularly among women. These facts suggest that a plausible neighborhood socioeconomic transformation may be linked to improvements in decrease the death by suicide. Meanwhile, no correlation was found between gentrification and homicide rates, suggesting that complex structural determinants underlie homicide among Mexican Citizens.

## Data Availability

Publicly available datasets were analyzed in this study. Data from all the municipalities of Mexico City were included. The data are free of the Attorney General’s Office of Mexico City (Mexico, 2025).
